# Automated titanium fastener for surgical aortic valve replacement—preventive role for infective endocarditis?

**DOI:** 10.1093/ejcts/ezae236

**Published:** 2024-06-24

**Authors:** Amila Kahrovic, Harald Herkner, Philipp Angleitner, Paul Werner, Alfred Kocher, Marek Ehrlich, Dominik Wiedemann, Guenther Laufer, Paul Simon, Martin Andreas

**Affiliations:** Department of Cardiac Surgery, Medical University of Vienna, Vienna, Austria; Department of Emergency Medicine, Medical University of Vienna, Vienna, Austria; Department of Cardiac Surgery, Medical University of Vienna, Vienna, Austria; Department of Cardiac Surgery, Medical University of Vienna, Vienna, Austria; Department of Cardiac Surgery, Medical University of Vienna, Vienna, Austria; Department of Cardiac Surgery, Medical University of Vienna, Vienna, Austria; Department of Cardiac Surgery, Medical University of Vienna, Vienna, Austria; Department of Cardiac Surgery, Medical University of Vienna, Vienna, Austria; Department of Cardiac Surgery, Medical University of Vienna, Vienna, Austria; Department of Cardiac Surgery, Medical University of Vienna, Vienna, Austria

**Keywords:** Automated titanium fastener, Hand-tied knots, Surgical aortic valve replacement, Infective endocarditis

## Abstract

**OBJECTIVES:**

Evidence on long-term clinical outcomes considering suture-securing techniques used for surgical aortic valve replacement is still uncertain.

**METHODS:**

A total of 1405 patients who underwent surgical aortic valve replacement between January 2016 and December 2022 were included and grouped according to the suture-securing technique used (automated titanium fastener versus hand-tied knots). The occurrence of infective endocarditis during follow-up was set as the primary study end-point. As secondary study end-points, stroke, all-cause mortality and a composite outcome of either infective endocarditis, stroke, or all-cause mortality were assessed.

**RESULTS:**

The automated titanium fastener was used in 829 (59%) patients, whereas the hand-knot tying technique was used in 576 (41%) patients. The multivariable proportional competing risk regression analysis showed a significantly lower risk of infective endocarditis during follow-up in the automated titanium fastener group (adjusted sub-hazard ratio 0.44, 95% confidence interval 0.20–0.94, *P* = 0.035). The automated titanium fastener group was not associated with an increased risk of mortality or attaining the composite outcome, respectively (adjusted hazard ratio 0.81, 95% confidence interval 0.60–1.09, *P* = 0.169; adjusted hazard ratio 0.82, 95% confidence interval 0.63–1.07, *P* = 0.152). This group was not associated with an increased risk of stroke (adjusted sub-hazard ratio 0.82, 95% confidence interval 0.47–1.45, *P* = 0.504). Also, a significantly lower rate of early-onset infective endocarditis was observed in the automated titanium fastener group, (0.4% vs 1.4%, *P* = 0.032).

**CONCLUSIONS:**

Suture-securing with an automated titanium fastener device appears to be superior compared to the hand-knot tying technique in terms of lower risk of infective endocarditis.

## Graphical abstract

**Figure:**
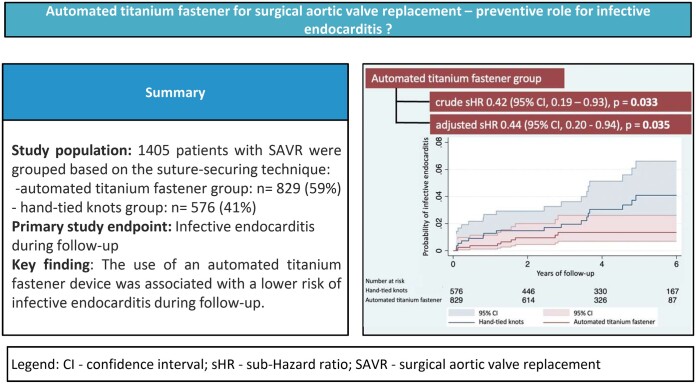

## INTRODUCTION

Surgical aortic valve replacement (SAVR) is a routinely performed procedure to treat different pathologies of the aortic valve, including aortic valve stenosis, aortic valve regurgitation or a combination of both stenosis and regurgitation. When indicated, SAVR can be performed with either biological or mechanical valve prosthesis [1].

In the context of aortic valve prosthesis fixation, sutures can be secured using an automated titanium fastener device (Cor-Knot Device, LSI Solutions, Victor, New York, USA) or a conventional technique of hand-knot tying. The automated titanium fastener device is designed to facilitate suture-securing through pivotal steps including the advancement of the device over slightly tensioned suture to the designated position, then the lever of the device is squeezed, releasing the crimped automated titanium fastener, and simultaneously trimming excess suture tails [2].

The objectives of this study were to comprehensively analyse the long-term clinical outcomes of patients who underwent SAVR with respect to the suture-securing technique (automated titanium fastener versus hand-tied knots).

## PATIENTS AND METHODS

### Ethical statement

The Ethics Committee of the Medical University Vienna approved this study, in compliance with the Declaration of Helsinki; (vote number 1243/2023; approval date: 8 May 2023). Informed consent was waived.

### Study design

A total of 2465 consecutive adult patients who underwent SAVR between January 2016 and December 2022 at the Department of Cardiac Surgery, Medical University of Vienna were screened for inclusion and exclusion criteria. The exclusion criteria were surgery of mitral, tricuspid or pulmonary valve in combination with SAVR, Bentall procedure, replacement of ascending aorta, previous cardiac surgery and infective endocarditis (IE) at baseline; Supplementary Material, Fig. S1. One thousand four hundred five patients were considered as eligible for inclusion in this retrospective study and grouped according to the suture-securing technique used (automated titanium fastener versus hand-tied knots) during SAVR. Identification of suture-securing technique relied on a comprehensive review of operation reports and institutional surgery protocols. The decision to use either suture-securing technique was driven by the individual preferences of the operating surgeon.

### Study endpoints and definitions

The occurrence of IE during follow-up was set as the primary study endpoint. As secondary endpoints, the occurrence of stroke, all-cause mortality and a composite outcome (defined as the occurrence of either IE, stroke or all-cause mortality) were assessed. The diagnosis of IE was defined following the modified Duke criteria [3], irrespective of whether surgical or conservative treatment was pursued. The occurrence of IE of the aortic valve prosthesis was categorized as early-onset, if the infection developed within the initial 12 months of SAVR and as late-onset, if it occurred after 1 year [3]. Stroke was defined as the manifestation of a prolonged or permanent neurological impairment, confirmed by the acute lesion on a neuroimaging scan [4]. All-cause mortality encompassed all deaths following SAVR, regardless of the underlying cause. Survival data were retrieved from federal statistics (Statistics Austria). Study endpoints were extracted from patients’ medical records. A complete dataset was generated using a prospectively managed database at our institution. A total of 4.6% (*n* = 64) patients were considered as lost to follow-up. The follow-up period was terminated upon the occurrence of each study endpoint, the death of the patient, the last date of hospital visit for those categorized as lost to follow-up, or upon the end of the study. The last date of follow-up was 28 September 2023.

### Statistical analysis

Data are presented as median and interquartile range (IQR) for continuous variables, and frequencies and percentages for categorical variables. Statistical calculations comparing continuous variables were made with the Mann–Whitney *U*-test for not normally distributed variables. Comparisons of categorical variables were made using the chi-square test. To evaluate the crude and adjusted effect of the suture-securing techniques (automated titanium fastener versus hand-tied knots) on the study endpoints, univariable and multivariable proportional hazards regression analyses were performed. The exposure time started from the date of the surgery. The selection of co-variables for the multivariable models was based on a causal concept, considering published evidence and clinical experience.

The multivariable proportional subhazards Fine and Gray regression model accounting for death as a competing event was used to analyse the effect of the suture-securing technique on IE during follow-up. The estimated subhazard ratios (sHR) with 95% confidence intervals (CI) were provided. The following co-variables were included in the model: age (years^2^), body mass index (kg/m^2^), dialysis, bicuspid aortic valve, urgent surgery, full sternotomy, biological valve prosthesis and coronary artery bypass grafting. As a sensitivity analysis, the influence of suture-securing technique type on the risk of IE was assessed using inverse-probability-weighted regression adjustment. However, the results of this model were in agreement with those from multivariable proportional competing risk regression model and therefore not reported.

To quantify the model performance, we calculated Harrel’s C, where possible. Based on statistical metrics, the multivariable regression model was deemed appropriate. Kaplan–Meier cumulative event curves were generated to visualize the probability of IE between the study groups. Additionally, we tested for an effect modification of the main effect by different aortic valve prostheses (valve-specific sub-groups) by including an interaction term into the models.

For the secondary study endpoint, stroke, the multivariable proportional subhazards Fine and Gray regression analysis accounting for death as a competing event was used. We selected the following co-variables in the model: era (2016–2019 years), age (years^2^), history of stroke, cerebrovascular disease, atrial fibrillation, dialysis and biological valve prosthesis.

Furthermore, the multivariable Cox proportional hazards regression models were generated to investigate the effects of the suture-securing technique on all-cause mortality and composite outcome. The effects were reported as an adjusted hazard ratio (HR) with 95% CI. In the analysis of all-cause mortality, the model was adjusted for European System for Cardiac Operative Risk Evaluation II (EuroSCORE II) (log-transformed), era (2016–2019 years), body mass index (kg/m^2^), biological valve prosthesis and cardiopulmonary bypass time (min). For the assessment of the composite outcome, the model included the following co-variables: era (2016–2019 years), age (years^2^), EuroSCORE II (log-transformed), diabetes mellitus, dialysis, cerebrovascular disease and biological valve prosthesis.

The statistical analysis was performed using SPSS software 27.0 (IBM Corp, Armonk, NY, USA) and STATA 16.1 software (StataCorp LLC, College Station, TX, USA). A two-sided *P*-value of <0.05 was considered as statistically significant.

## RESULTS

### Baseline characteristics

The automated titanium fastener was used in 829 patients (59%), while the hand-knot tying technique was performed in 576 patients (41%). Patients in the automated titanium group were younger (median 70.5, IQR 62.4–76.2 versus median 72.1, IQR 64.1–77.5, *P* = 0.007) and were presented with a higher body mass index (median 28.0, IQR 25.3–31.7 versus median 27.5, IQR 24.8–30.9, *P* = 0.034; Table 1). All other baseline characteristics variables were comparable between study groups. Preoperative echocardiography data are presented in the Supplementary Material, Table S5. In terms of aortic valve anatomy, the automated titanium fastener group was presented with a lower rate of a tricuspid aortic valve (65.9% vs 76.0%, *P* < 0.001), and a higher rate of a bicuspid aortic valve (32.6% vs 22.7%, *P* < 0.001).

**Table 1:** Baseline characteristics

Variables	Automated titanium fastener, *N* = 829 (59%)	Hand-tied knots, *N* = 576 (41%)	*P*-value
Age (years) (25th–75th interval)	70.5 (62.4–76.2)	72.1 (64.1–77.5)	**0.007**
Female (%)	271 (32.7)	211 (36.6)	0.126
Body mass index (kg/m^2^) (25th–75th interval)	28.0 (25.3–31.7)	27.5 (24.8–30.9)	**0.034**
EuroSCORE II, 25th–75th interval	1.8 (1.1–3.4)	2.1 (1.1–3.6)	0.054
NYHA class III–IV, *n* (%)	300 (36.2)	230 (39.9)	0.155
Hypertension, *n* (%)	726 (87.6)	489 (84.9)	0.149
Atrial fibrillation, *n* (%)	136 (16.4)	96 (16.7)	0.897
Paroxysmal	102 (12.3)	69 (12.0)	0.855
Persistent	34 (4.1)	27 (4.7)	0.596
Previous pacemaker implantation, *n* (%)	25 (3.0)	19 (3.3)	0.765
Diabetes mellitus, *n* (%)	232 (28.0)	160 (27.8)	0.932
Peripheral vascular disease, *n* (%)	84 (10.1)	46 (8.0)	0.172
Cerebrovascular disease, *n* (%)	161 (19.4)	93 (16.1)	0.117
History of stroke, *n* (%)	42 (5.1)	24 (4.2)	0.433
Dialysis, *n* (%)	6 (0.7)	8 (1.4)	0.217

Bold indicates statistical significance (*P* < 0.05).

EuroSCORE II: European System for Cardiac Operative Risk Evaluation II; NYHA: New York Heart Association.

### Operative characteristics

In the 1st study era (2016–2019), the hand-knot tying technique was used more frequently (75.9%) compared to automated titanium fastener (51.5%). However, in the subsequent era (2020–2022), there was a significant shift towards increased use of automated titanium fastener, (48.6% vs 24.1%, *P* ≤ 0.001; Table 2). The automated titanium fastener group underwent urgent SAVR at a significantly lower rate (10.5% vs 14.1%, *P* = 0.043). Concerning surgical access, this group had a lower rate of full-sternotomy (47.4% vs 59.7%, *P* < 0.001) and a higher rate of hemi-sternotomy and thoracotomy, respectively (36.8% vs 31.4%, *P* = 0.037; 15.8% vs 8.9%, *P* ≤ 0.001). No differences were found among the study groups regarding the use of mechanical or biological prostheses. As a concomitant procedure, coronary artery bypass grafting was performed at a lower rate in the automated titanium fastener group (31.7% vs 41.0%, *P* ≤ 0.001). Details on implanted aortic valve prostheses are provided in Supplementary Material, Table S6.

**Table 2:** Operative characteristics

Variables	Automated titanium fastener, *N* = 829 (59%)	Hand-tied knots, *N* = 576 (41%)	*P*-value
Era, *n* (%)			**<0.001**
2016–2019	426 (51.5)	437 (75.9)	
2020–2022	403 (48.6)	139 (24.1)	
Urgency status, *n* (%)			
Elective	729 (87.9)	487 (84.5)	0.067
Urgent	87 (10.5)	81 (14.1)	**0.043**
Emergency	13 (1.6)	8 (1.4)	0.785
Salvage	0 (0.0)	0 (0.0)	–
Access, *n* (%)			
Full-sternotomy	393 (47.4)	344 (59.7)	**<0.001**
Hemi-sternotomy	305 (36.8)	181 (31.4)	**0.037**
Thoracotomy	131 (15.8)	51 (8.9)	**<0.001**
Aortic valve replacement, *n* (%)			0.830
Mechanical prosthesis	101 (12.2)	68 (11.8)	
Biological prosthesis	728 (87.8)	508 (88.2)	
Concomitant procedures, *n* (%)			
CABG	263 (31.7)	236 (41.0)	**<0.001**
Aortic reduction plasty	81 (9.8)	62 (10.8)	0.545
Aortic root enlargement	27 (3.3)	24 (4.2)	0.370
Septal myectomy	46 (5.5)	34 (5,9)	0.778
Atrial fibrillation surgery	46 (5.5)	36 (6.3)	0.581
Left atrial appendage resection	58 (7.0)	54 (9.4)	0.105
CPB time (min), 25th–75th interval	116 (95–148)	115 (92–149)	0.134
Total operating room time (min), 25th–75th interval	245 (203–298)	250 (203–300)	0.613

Bold indicates statistical significance (*P* < 0.05).

CABG: Coronary artery bypass grafting; CPB: cardiopulmonary bypass.

### Postoperative adverse events

No differences were found among the study groups, regarding postoperative adverse events; Supplementary Material, Table S7.

### Long-term outcomes

The rate of ischaemic and haemorrhagic stroke during follow-up was similar between the study groups (Table 3). The rate of reintervention was lower in the automated titanium fastener group (1.3% vs 3.5%, *P* = 0.007). Indications for reintervention included paravalvular leak, structural valve deterioration and IE. No statistically significant difference was observed in the rate of reoperation due to paravalvular leak between the automated titanium fastener group and the hand-tied knots group (0.4% vs 1.0%, *P* = 0.116). Irrespective of the chosen therapeutic approach (surgery or no surgery), IE of the aortic valve prosthesis occurred less frequently in the automated titanium fastener group (1.1% vs 3.0%, *P* = 0.011). Also, this group had a significantly lower rate of early-onset IE (0.4% vs 1.4%, *P* = 0.032). Concerning other reoperations or transcatheter procedures during follow-up, no differences were found among the study groups (Supplementary Material, Table S8).

**Table 3:** Long-term outcomes

Variables	Automated titanium fastener, *N* = 829 (59%)	Hand-tied knots, *N* = 576 (41%)	*P*-value
Stroke			
Ischaemic, *n* (%)	22 (2.7)	21 (3.6)	0.288
Haemorrhagic, *n* (%)	5 (0.6)	3 (0.5)	0.840
Reintervention, *n* (%)	11 (1.3)	20 (3.5)	**0.007**
Paravalvular leak	3 (0.4)	6 (1.0)	0.116
Structural valve deterioration	3 (0.4)	5 (0.9)	0.215
Valve thrombosis	0 (0.0)	0 (0.0)	–
Infective endocarditis (surgery)	5 (0.6)	9 (1.6)	0.075
Infective endocarditis (no surgery), *n* (%)	4 (0.5)	8 (1.4)	0.069
Infective endocarditis (all cases), *n* (%)	9 (1.1)	17 (3.0)	**0.011**
Early-onset	3 (0.4)	8 (1.4)	**0.032**
Late-onset	6 (0.7)	9 (1.6)	0.132

Bold indicates statistical significance (*P* < 0.05).

### Study endpoints

#### Primary study endpoint

**Figure 1:**
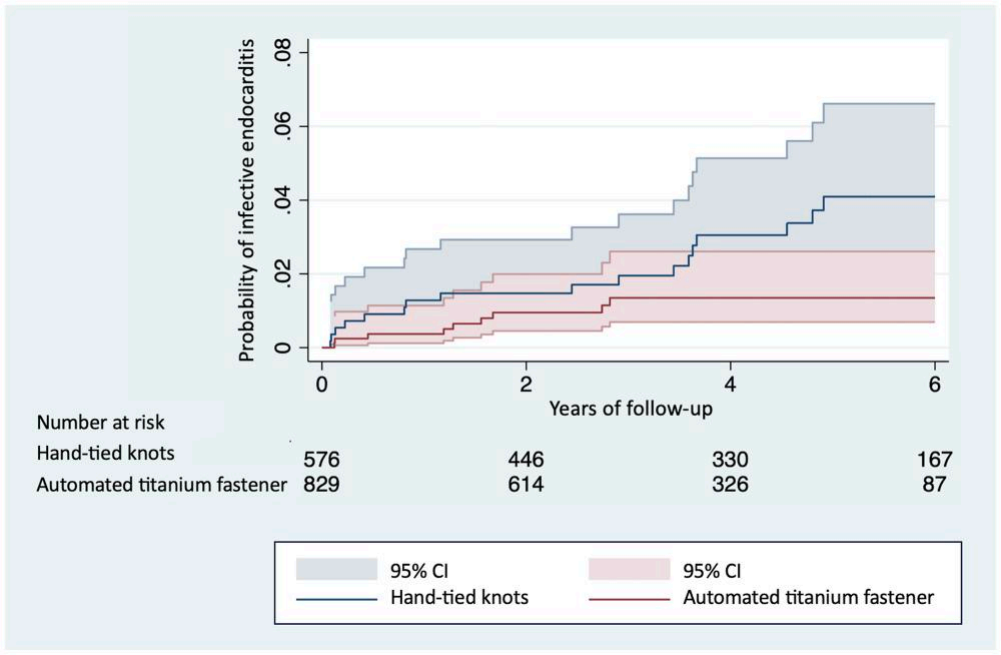
Kaplan–Meier cumulative event curves show the probability of IE between the automated titanium fastener group versus hand-tied knots group. CI: confidence interval; IE: infective endocarditis.

The risk of IE during follow-up was significantly lower in the automated titanium fastener group, as indicated in the univariable proportional competing risk regression analysis (sHR 0.42, 95% CI 0.19–0.93, *P* = 0.033; Table 4). Additionally, the multivariable proportional competing risk regression analysis demonstrated a significantly lower risk of IE in this group (adjusted sHR 0.44, 95% CI 0.20–0.94, *P* = 0.035; Table 4; Supplementary Material, Table S1). The primary study endpoint was visually represented by the Kaplan–Meier cumulative event curves of both study groups (Fig. 1). The effect was not modified by different aortic valve prostheses (valve-specific sub-groups) in any of the interaction analyses. The median follow-up duration was 1239 days (IQR 706–1829) for the automated titanium fastener group and 1686 days (IQR 842–2309) for the hand-tied knots group.

**Table 4:** Study endpoints

	Automated titanium fastener, *N* = 829 (59%)	Hand-tied knots, *N* = 576 (41%)	Univariable relative effects		Multivariable relative effects	
			95% CI	*P*-value	95% CI	*P*-value
Infective endocarditis^a^	9 (1.1)	17 (3.0)	0.42 (0.19–0.93)	**0.033**	0.44 (0.20–0.94)	**0.035**
Stroke^a^	27 (3.3)	24 (4.2)	0.84 (0.48–1.45)	0.524	0.82 (0.47–1.45)	0.504
All-cause mortality^b^	86 (10.4)	100 (17.4)	0.76 (0.57–1.01)	0.063	0.81 (0.60–1.09)	0.169
Composite outcome^b^	109 (13.1)	124 (21.5)	0.74 (0.57–0.96)	**0.024**	0.82 (0.63–1.07)	0.152

Bold indicates statistical significance (*P* < 0.05).

a Effects calculated as sHR based on multivariable proportional competing risk regression model.

b Effects calculated as HR based on multivariable Cox proportional hazards regression model.

CI: confidence interval; HR: hazard ratio; sHR: sub-hazard ratio.

#### Secondary study endpoints

The use of an automated titanium fastener was not associated with an increased risk of stroke during follow-up, as evidenced in the multivariable proportional competing risk regression analysis (adjusted sHR 0.82, 95% CI 0.47–1.45, *P* = 0.504; Table 4; Supplementary Material, Table S2). Notably, older age and cerebrovascular disease at baseline were independently associated with an increased risk of stroke, respectively (adjusted sHR 1.00, 95% CI 1.00–1.01, *P* = 0.009; adjusted sHR 2.04, 95% CI 1.10–3.79, *P* = 0.024; Supplementary Material, Table S2).

Overall, 186 patients (13.2%) died during follow-up. The multivariable Cox proportional hazards regression model showed that the use of automated titanium fastener was not associated with an increased risk of all-cause mortality (adjusted HR 0.81, 95% CI 0.60–1.09, *P* = 0.169) (Table 4; Supplementary Material, Table S3).

The risk of experiencing the composite outcome was significantly lower in the automated titanium fastener group based on the univariable Cox proportional hazards regression analysis (adjusted HR 0.74, 95% CI 0.57–0.96, *P* = 0.024; Table 4). Further, in the multivariable Cox proportional hazards regression model, the automated titanium fastener group was not associated with an increased risk of attaining the composite outcome during follow-up (adjusted HR 0.82, 95% CI 0.63–1.07, *P* = 0.152; Table 4; Supplementary Material, Table S4).

### Clinical presentation of patients presenting with infective endocarditis during the follow-up

A total of 26 patients (1.9%), identified during the follow-up period, met the criteria for definite IE diagnosis, in accordance with the modified Duke criteria and clinical guidelines [3] (Supplementary Material, Figs S2 and S3). Prevalence rates of major and minor criteria are presented in Supplementary Material, Table S9. Furthermore, Supplementary Material, Table S10 lists the pathogens detected in the blood culture results and intraoperative smears (if applicable—patients who underwent surgery for IE).

### Causes of death

Cardiac-related mortality emerged as the predominant cause of death, with respiratory causes and multiorgan failure following in succession (Table 5).

**Table 5:** Causes of death

Variables	Automated titanium fastener, *N* = 829 (59%)	Hand-tied knots, *N* = 576 (41%)	*P*-value
Cardiac, *n* (%)	34 (4.1)	31 (5.4)	0.261
Respiratory, *n* (%)	13 (1.6)	18 (3.1)	0.051
COVID-19	6 (0.7)	10 (1.7)	0.079
Multi-organ failure, *n* (%)	14 (1.7)	13 (2.3)	0.445
Infection, *n* (%)	5 (0.6)	8 (1.4)	0.130
Cancer, *n* (%)	7 (0.8)	8 (1.4)	0.329
Neurological, *n* (%)	3 (0.4)	7 (1.2)	0.061
Other, *n* (%)	4 (0.5)	5 (0.9)	0.373
Unknown, *n* (%)	6 (0.7)	10 (1.7)	0.079

COVID-19: Coronavirus disease 2019.

## DISCUSSION

This study represents the 1st comparative analysis of long-term clinical outcomes among a cohort of 1405 patients, with a focus on the suture-securing technique used (automated titanium fastener versus hand-tied knots) during SAVR. The major finding of the study is that the use of an automated titanium fastener device was associated with a lower risk of IE during follow-up (sHR 0.44, 95% CI 0.20–0.94, *P* = 0.035).

**Figure 2:**
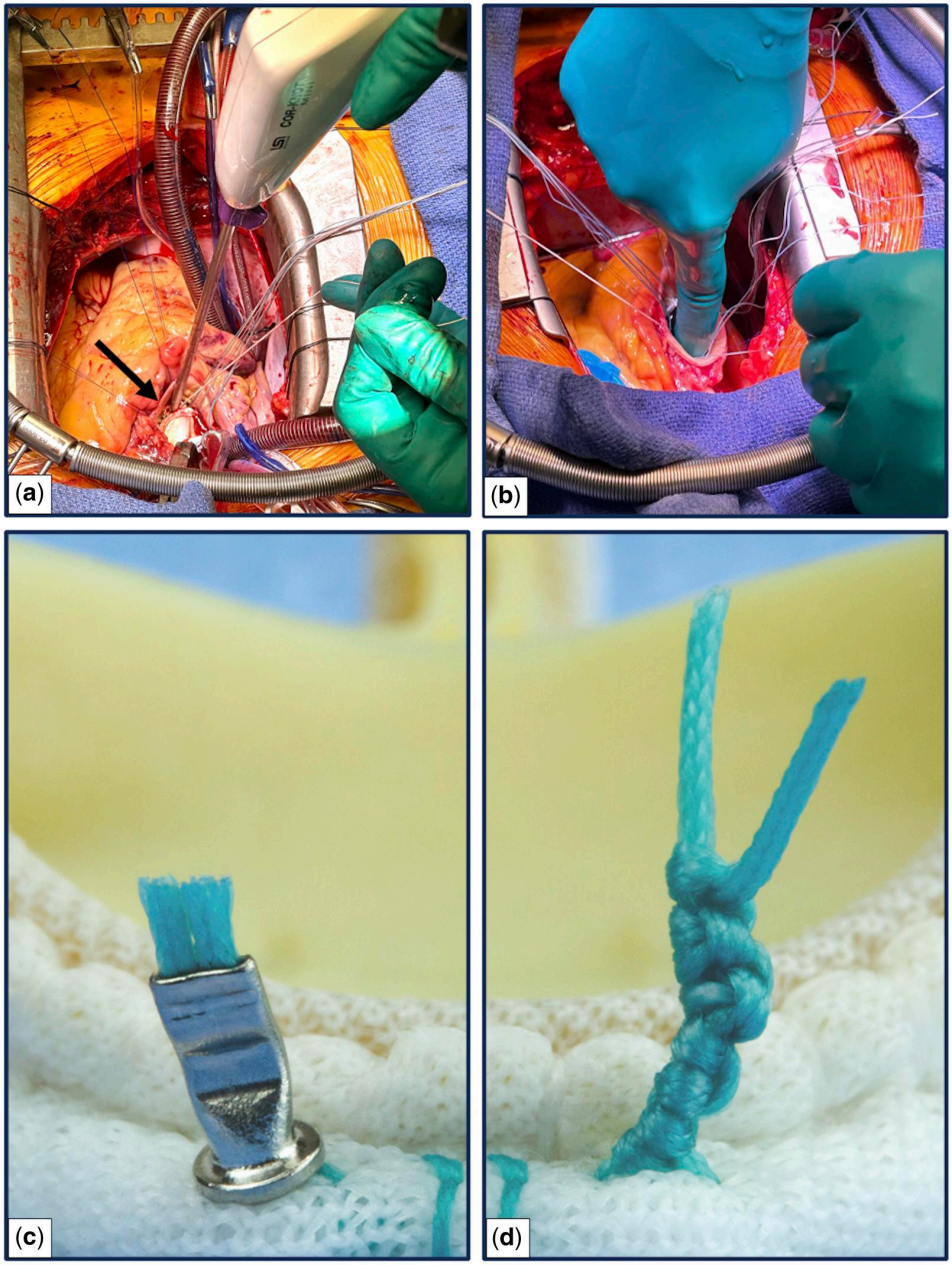
Suture-securing techniques during SAVR (automated titanium fastener versus hand-tied knots). (a) Deployment of an automatic titanium fastener with no direct hand-contact on the newly implanted aortic valve prosthesis. The arrow indicates the position of the automatic titanium fastener device at the target site. (b) Tying of the knot manually, with the focus on direct hand-contact on the newly implanted aortic valve prosthesis. (c) Enlarged view of the flat, smooth, uniform surface of automated titanium fastener crimped around the suture. (d) Enlarged representation of hand-tied knots with numerous cervices and throws for potential bacterial attachment. The total number of throws amounts to 8. SAVR: surgical aortic valve replacement.

One of the primary advantages of employing an automated titanium fastener device might be the potential reduction in contamination through the implementation of the no-touch principle. Importantly, the suture-securing with an automated titanium fastener device is achieved through a single deployment, with no direct hand-contact with the newly implanted valve prosthesis (Fig. 2a). In contrast, the hand-knot tying technique involves multiple, direct hand-touches for securing 1 suture, resulting in over a hundred direct hand-touches on the newly implanted valve prosthesis (Fig. 2b). Following extended surgical procedure, it is pertinent to note that surgical gloves might bear pathogens and subsequently, disseminate these while tying knots manually [5]. Surgical site contamination, including the newly implanted prosthesis, might lead to postoperative occurrence of early-onset IE [6]. Indeed, among the pathogens detected in the blood culture results of patients presenting with IE during follow-up (Supplementary Material, Table S10), several belong to the normal flora of the skin, including *Staphylococci* and *Streptococci* species [7].

Secondary, surface characteristics should not be underestimated, in the context of bacterial attachment [8, 9]. Irregularities in topography, as observed in braided sutures with multiple knots, introduce numerous microenvironments where bacteria can adhere and thrive [10, 11] (Fig. 2d). Also, multiple tied, braided sutures may provide an increased surface area for bacterial attachment compared to the smoother, flatter surface of an automated titanium fastener [11, 12]. The establishment of the bacterial attachment, either due to contamination or during transient bacteraemia, is an initial stage in the development of an infection [13]. Following bacterial adherence to the surface, bacteria tend to multiply in a more organized and cooperative manner within an extracellular polysaccharide slime-like matrix, forming a structured biofilm. The biofilm creates a protective environment that allows bacteria to grow, impede antibiotic efficacy and the host’s immune defences, as well as exhibit increased virulence [8, 13]. Conversely, the flat, uniform surface of the automated titanium fastener, crimped around the suture might be less conducive to bacterial adherence (Fig. 2c).

Additionally, a significantly lower rate of early-onset IE of the aortic valve prosthesis was observed in the automated titanium fastener group (0.4% vs 1.4%, *P* = 0.032). Concerning the pathogenesis of early-onset IE, in instances of contamination, infection usually involves braided sutures and the sewing ring, expanding to the annulus, and resulting in the formation of IE-related lesions (abscesses, pseudoaneurysms and fistulae), as stated in the 2023 European Society of Cardiology Guidelines for the Management of Infective Endocarditis [3].

The current analysis indicates that the automated titanium fastener was not associated with an increased risk of stroke during follow-up (adjusted sHR 0.82, 95% CI 0.47–1.45, *P* = 0.504). Several studies reported no difference in the rate of short-term stroke onset among the study groups [14, 15]. Indeed, surgical titanium is a commonly used material in medical devices, primarily due to its favourable mechanical properties and biocompatibility. Although surgical titanium may have thrombogenic potential, the subsequent neo-endothelialization of the surface might enhance its haemocompatibility, thus mitigating the risk of blood clot formation or other adverse events, such as haemolysis [16, 17]. Furthermore, Li *et al.* conducted an experimental study using an ovine model to assess the biocompatibility, local tissue pathological changes, inflammatory response and thrombosis tendency of an automated titanium fastener compared to hand-tied knots. The study found that neo-endothelialization was comparable among the study groups by day 60. Notably, no thrombus formation was detected on the surface of the automated titanium fastener [18].

Concerning mortality, the systematic review and meta-analysis examined mortality between the automated titanium fastener group and the hand-tied knots group, indicating no significant difference [19], which is consistent with our findings.

Suture-securing with an automated titanium fastener provides uniform tension along the prosthesis [2]. An experimental study by Lee *et al.* demonstrated that sutures secured with automated titanium fastener are significantly more consistent, on average twice as strong as hand-tied knots, which might reduce the risk of paravalvular leak formation [4]. Indeed, the occurrence of the paravalvular leak might have a technical nature and might be attributed to the undesirable loose hand-tied knots or ‘air-knots’. In the present analysis, the rate of reoperation due to the paravalvular leak was lower in the automated titanium fastener group; however, the significance level has not been reached (0.4% vs 1.0%, *P* = 0.116).

The cases of leaflet perforation attributed to automated titanium fastener have been reported in a few case reports [20]. To ensure safe deployment of the automated titanium fastener, the suture slot and the rotational knob’s indicator fin should be consistently aligned towards the centre of the prosthesis. Noteworthy, in the present study involving 829 patients in the automated titanium fastener group, no such complication was documented.

In terms of economics, the cost of the automated titanium fastener device is ∼€165 for the European market, with an additional charge of ∼€45 for each individual loading unit used for the fixation of a single stitch. The financial implication of implementing such technology is essential for healthcare institutions as it may infuence decision-making processes.

### Limitations

The present analysis is a single-centre retrospective study. Only patients who underwent isolated SAVR were included in the study, which may not represent the real patient population. Given the retrospective nature of the study, notable selection bias is apparent, manifested in the disparity in age, era, aortic valve anatomy, surgical access, urgent setting and concomitant coronary artery bypass grafting surgery. The use of either suture-securing technique was surgeon-dependent. The number of stitches utilized for prosthesis fixation was variable though different types and sizes of aortic valve prostheses. Information regarding minor paravalvular leak, as the possible predisposition for IE, was not available for statistical analysis. The occurrence of IE during follow-up can be caused by several factors, independent of the suture-securing technique used. Only patients with a definite diagnosis of IE (following modified Duke criteria and current guidelines [3]) were considered for the analysis. The current literature on this topic is limited.

## CONCLUSIONS

The automated titanium fastener device seems to be a promising surgical tool not only for facilitating SAVR, but also potentially reducing the risk of IE compared to the hand-knots tying technique. Avoiding direct hand-contact on the valve prosthesis by implementing the ‘no-touch principle’ with an automated titanium fastener device in valve surgery might be beneficial. Also, the importance of suture characteristics for potential bacterial adherence should not be underestimated. A prospective randomized controlled trial is warranted to confirm our findings.

## Supplementary Material

ezae236_Supplementary_Data

## Data Availability

Anonymized datasets will be shared on reasonable request to the corresponding author.

## Notes

The abstract has been submitted to the 38th EACTS Annual Meeting.

## ABBREVIATIONS

CI: Confidence interval
EuroSCORE II: European System for Cardiac Operative Risk Evaluation II
HR: Hazard ratio
IE: Infective endocarditis
IQR: Interquartile range
SAVR: Surgical aortic valve replacement
sHR: Subhazard ratio
