# Supplementary material for: Automated titanium fastener for surgical aortic valve replacement—preventive role for infective endocarditis?
Source: Eur J Cardiothorac Surg. 2024 Jun 24;65(6):ezae236. doi: 10.1093/ejcts/ezae236 (PMC11211209; doi:10.1093/ejcts/ezae236)
Supplement: ezae236_Supplementary_Data [file ezae236_supplementary_data.docx]

**Supplemental figure S1.** Study flowchart; SAVR: surgical aortic valve replacement


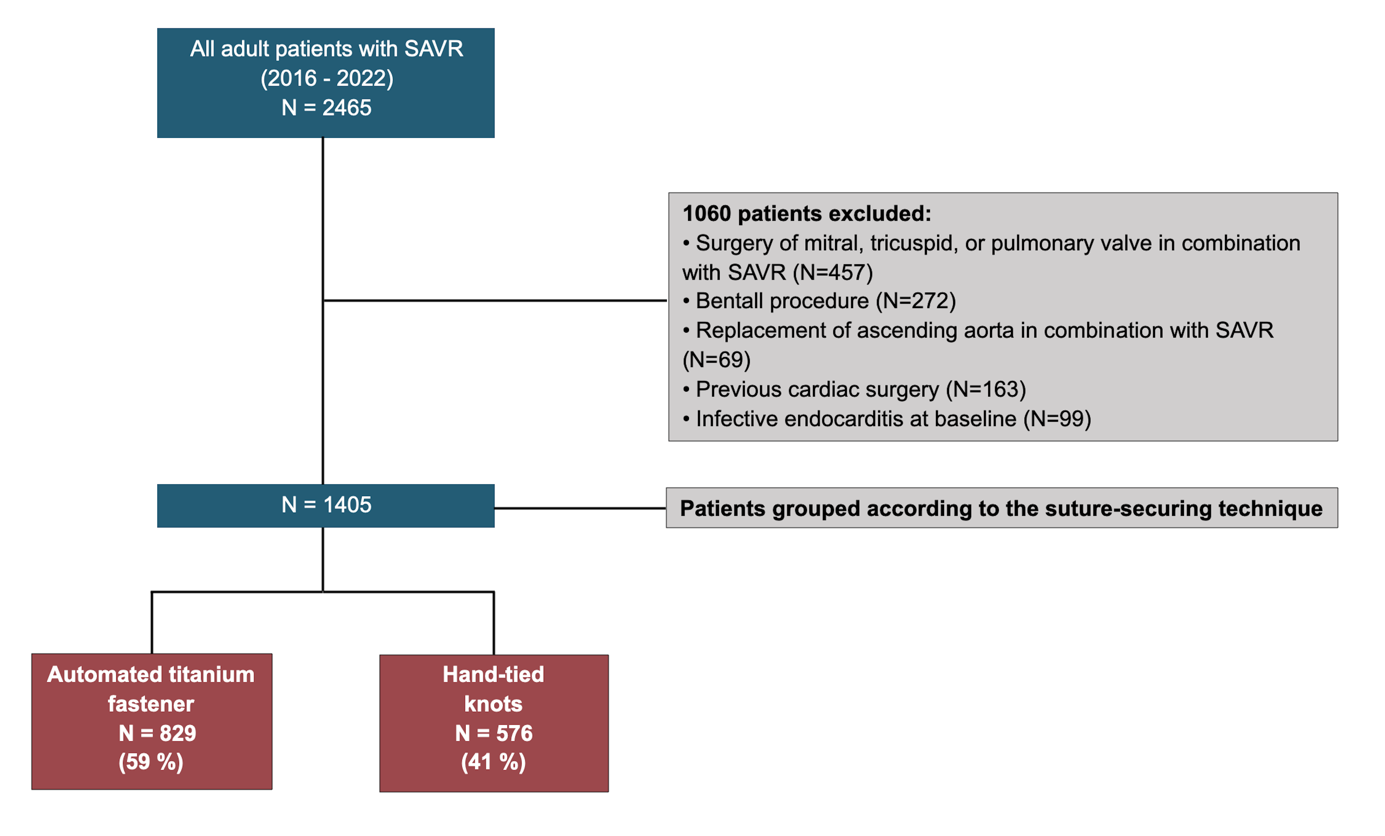


**Supplemental figure S2.** Diagnostic modified Duke criteria for each patient presenting with IE during follow-up in the automated titanium fastener group. IE: infective endocarditis**
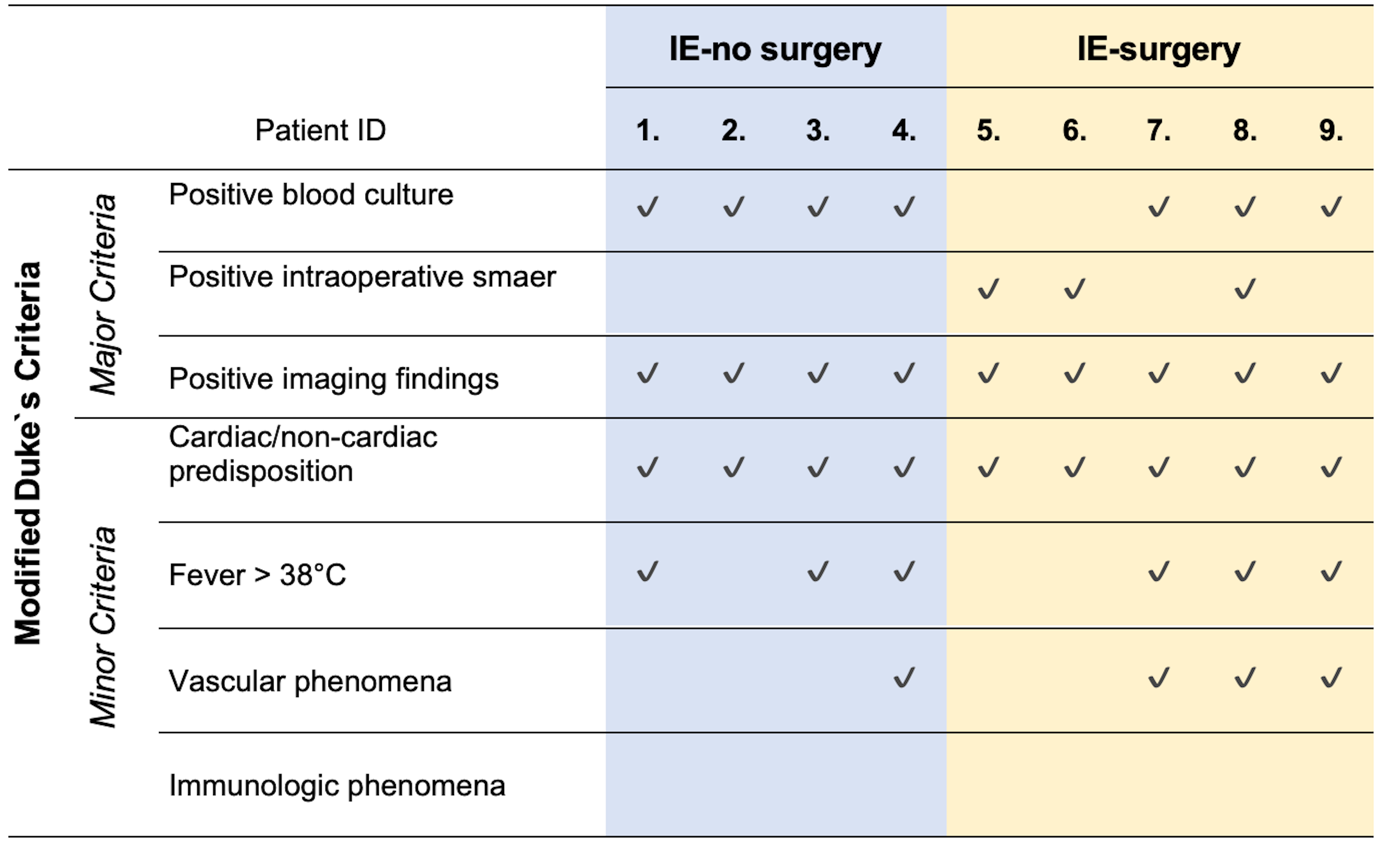
**

**Supplemental figure S3.** Diagnostic modified Duke criteria for each patient presenting with IE during follow-up in the hand-tied knots group. IE: infective endocarditis

**
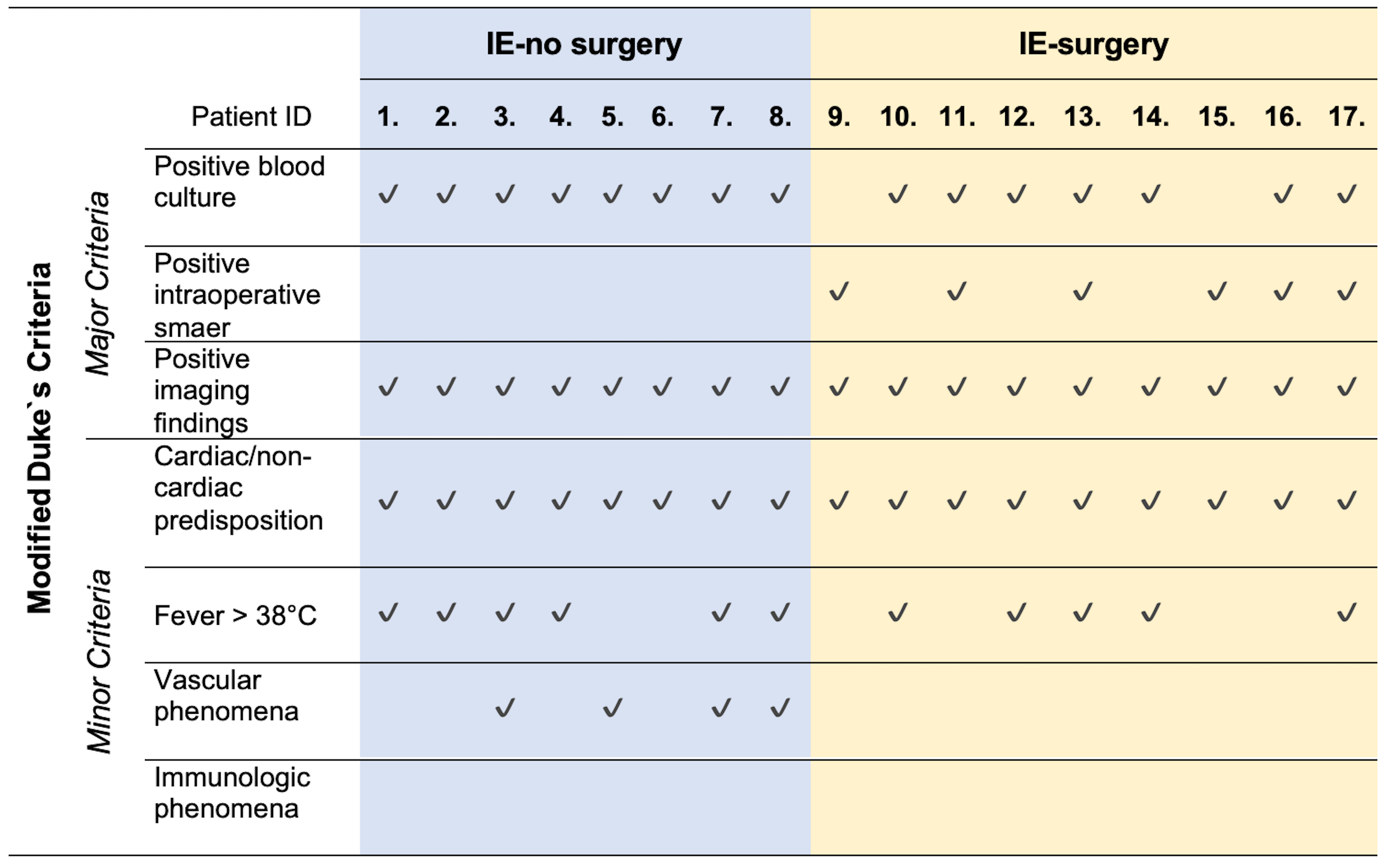
**

**Supplemental table S1: Primary study endpoint – Infective endocarditis ^a^**

| **Variables** | **sHR** | **95% CI** | **p-value** |
| --- | --- | --- | --- |
| Automated titanium fastener | 0.44 | 0.20 – 0.94 | **0.035** |
| Age (years^2^) | 1.00 | 1.00 – 1.00 | 0.836 |
| Body mass index (kg/m2) | 0.95 | 0.89 – 1.01 | 0.119 |
| Dialysis | 2.65 | 0.42 – 16.74 | 0.301 |
| Bicuspid aortic valve | 0.37 | 0.11 – 1.28 | 0.116 |
| Urgent surgery | 1.01 | 0.31 – 3.37 | 0.981 |
| Full sternotomy | 0.79 | 0.27 – 2.38 | 0.681 |
| Biological valve prosthesis | 2.77 | 0.32 – 24.13 | 0.357 |
| CABG | 0.84 | 0.26 – 2.66 | 0.762 |

Bold indicates statistical significance (p < 0.05).

a) Effects calculated as sHR based on multivariable proportional competing risk regression model.

CABG: Coronary artery bypass grafting; CI: confidence interval; sHR: subhazard ratio.

**Supplemental table S2: Secondary study endpoint – Stroke ^a^**

| **Variables** | **sHR** | **95% CI** | **p-value** |
| --- | --- | --- | --- |
| Automated titanium fastener | 0.82 | 0.47 – 1.45 | 0.504 |
| Era 2016 – 2019 years | 0.82 | 0.45 – 1.51 | 0.529 |
| Age (years^2^) | 1.00 | 1.00 – 1.01 | **0.009** |
| History of stroke | 0.65 | 0.15 – 2.77 | 0.560 |
| Cerebrovascular disease | 2.04 | 1.10 – 3.79 | **0.024** |
| Atrial fibrillation | 0.79 | 0.37 – 1.70 | 0.544 |
| Dialysis | 2.42 | 0.34 – 17.32 | 0.378 |
| Biological valve prosthesis | 0.51 | 0.17 – 1.49 | 0.218 |

Bold indicates statistical significance (p < 0.05).

a) Effects calculated as sHR based on multivariable proportional competing risk regression model.

CI: confidence interval; sHR: subhazard ratio.

**Supplemental table S3: Secondary study endpoint – All-cause mortality ^a^**

| **Variables** | **HR** | **95% CI** | **p-value** |
| --- | --- | --- | --- |
| Automated titanium fastener | 0.81 | 0.60 – 1.09 | 0.169 |
| EuroSCORE II (log-transformed) | 2.24 | 1.89 – 2.65 | **<0.001** |
| Era 2016 – 2019 years | 1.33 | 0.86 – 2.05 | 0.202 |
| Body mass index (kg/m2) | 1.04 | 1.01 – 1.07 | **0.005** |
| Biological valve prosthesis | 1.81 | 0.92 – 3.56 | 0.087 |
| CPB time (min) (25th–75th interval) | 1.00 | 1.00 – 1.01 | **0.018** |

Bold indicates statistical significance (p < 0.05).

a) Effects calculated as HR based on multivariable Cox proportional hazards regression model.

CI: confidence interval; CPB:cardiopulmonary bypass; EuroSCORE II: European System for Cardiac Operative Risk Evaluation II; HR: hazard ratio.

**Supplemental table S4: Secondary study endpoint – Composite outcome ^a^**

| **Variables** | **HR** | **95% CI** | **p-value** |
| --- | --- | --- | --- |
| Automated titanium fastener | 0.82 | 0.63 – 1.07 | 0.152 |
| Era 2016 – 2019 years | 1.15 | 0.80 – 1.65 | 0.444 |
| Age (years^2^) | 1.00 | 1.00 -1.00 | 0.430 |
| EuroSCORE II (log-transformed) | 1.90 | 1.60 – 2.25 | **<0.001** |
| Diabetes mellitus | 1.41 | 1.07 – 1.86 | 0.016 |
| Dialysis | 3.48 | 1.78 – 6.83 | **<0.001** |
| Cerebrovascular disease | 0.99 | 0.72 – 1.37 | 0.977 |
| Biological valve prosthesis | 1.26 | 0.66 – 2.40 | 0.490 |

Bold indicates statistical significance (p < 0.05).

a) Effects calculated as HR based on multivariable Cox proportional hazards regression model.

CI: confidence interval; EuroSCORE II: European System for Cardiac Operative Risk Evaluation II; HR: hazard ratio.

**Supplemental table S5: Preoperative echocardiography data**

| **Variables** | **Automated titanium fastener**  **N = 829**  **(59 %)** | **Hand-tied**  **knots**  **N = 576**  **(41 %)** | **p-value** |
| --- | --- | --- | --- |
| Left-ventricular function (%) |  |  |  |
| Good ≥50% | 631 (76.1) | 426 (74.0) | 0.357 |
| Moderate 31 – 50% | 167 (20.1) | 117 (20.3) | 0.939 |
| Poor 21 – 30% | 23 (2.8) | 22 (3.8) | 0.274 |
| Very poor ≤ 20% | 8 (1.0) | 11 (1.9) | 0.132 |
| Aortic valve stenosis (%) | 573 (69.1) | 407 (70.7) | 0.536 |
| Aortic valve regurgitation (%) | 70 (8.4) | 45 (7.8) | 0.671 |
| Combined aortic valve stenosis and | 186 (22.4) | 124 (21.5) | 0.686 |
| regurgitation (%) |  |  |  |
| Aortic valve anatomy (%) |  |  |  |
| Tricuspid | 546 (65.9) | 438 (76.0) | **<0.001** |
| Bicuspid | 270 (32.6) | 131 (22.7) | **<0.001** |
| Unicuspid | 13 (1.6) | 7 (1.2) | 0.583 |

Bold indicates statistical significance (p < 0.05).

**Supplemental table S6: Aortic valve prostheses implanted during SAVR**

| **Variables** | **Automated titanium fastener**  **N = 829**  **(59 %)** | | **Hand-tied**  **knots**  **N = 576**  **(41 %)** | **p-value** |
| --- | --- | --- | --- | --- |
| Aortic valve prostheses (%) |  | |  |  |
| CryoLife On-X Aortic | 98 (11.8) | | 59 (10.2) | 0.356 |
| Abbott Masters | 3 (0.4) | | 4 (0.7) | 0.384 |
| Medtronic ATS Medical | 0 (0.0) | | 5 (0.9) | **0.007** |
| Edwards Intuity | 274 (33.1) | | 297 (51.6) | **<0.001** |
| Edwards Inspiris | 204 (24.6) | | 63 (10.9) | **<0.001** |
| Edwards Magna Ease | 129 (15.6) | | 81 (14.1) | 0.438 |
| Medtronic Avalus | 88 (10.6) | | 10 (1.7) | **<0.001** |
| Medtronic Mosaic | 21 (2.5) | | 26 (4.5) | **0.042** |
| Abbott Trifecta | 9 (1.1) | | 25 (4.3) | **<0.001** |
| Mitroflow Sorin | 3 (0.4) | | 6 (1.0) | 0.116 |
| Implanted valve size (mm) (%) | |  |  |  |
| 19 | 35 (4.2) | | 29 (5.0) | 0.472 |
| 21 | 167 (20.1) | | 112 (19.4) | 0.746 |
| 23 | 262 (31.6) | | 167 (29.0) | 0.296 |
| 25 | 215 (25.9) | | 156 (27.1) | 0.631 |
| 27 | 127 (15.3) | | 98 (17.0) | 0.394 |
| 29 | 23 (2.8) | | 14 (2.4) | 0.692 |

Bold indicates statistical significance (p < 0.05).

SAVR: surgical aortic valve replacement

**Supplemental table S7: Postoperative adverse events**

| **Variables** | **Automated titanium fastener**  **N = 829**  **(59 %)** | **Hand-tied**  **knots**  **N = 576**  **(41 %)** | **p-value** |
| --- | --- | --- | --- |
| Atrial fibrillation (%) | 273 (32.9) | 217 (37.7) | 0.067 |
| Dialysis (%) | 18 (2.2) | 11 (1.9) | 0.734 |
| ECMO support (%) | 8 (1.0) | 5 (0.9) | 0.852 |
| New pacemaker implantation (%) |  |  |  |
| <14 days | 41 (4.9) | 41 (7.1) | 0.088 |
| 14 - 30 days | 3 (0.4) | 3 (0.5) | 0.653 |
| >30 - 60 days | 4 (0.5) | 2 (0.3) | 0.702 |
| Revision for bleeding (%) | 41 (4.9) | 27 (4.7) | 0.824 |
| Prolonged intensive care unit stay >7 days (%) | 62 (7.5) | 48 (8.3) | 0.558 |
| 30-day mortality (%) | 5 (0.6) | 6 (1.0) | 0.359 |

ECMO:  extracorporeal membrane oxygenation.

**Supplemental table S8: Other reoperations / Transcatheter procedures**

| **Variables** | **Automated titanium fastener**  **N = 829**  **(59 %)** | | | | **Hand-tied**  **knots**  **N = 576**  **(41 %)** | | **p-value** |
| --- | --- | --- | --- | --- | --- | --- | --- |
| Surgery/Transcatheter procedure of  mitral valve | | | | | | | |
| Mitral valve replacement (%) | | 1 (0.1) | | 0 (0.0) | | 0.404 | |
| Mitral valve repair (%) | | 1 (0.1) | | 0 (0.0) | | 0.404 | |
| TEER Mitral valve repair (%) | | 4 (0.5) | | 0 (0.0) | | 0.095 | |
| Surgery/Transcatheter procedure of  tricuspid valve | | | | | | | |
| Tricuspid valve replacement (%) | | 0 (0.0) | | 1 (0.2) | | 0.230 | |
| Tricuspid valve repair (%) | | 2 (0.2) | | 1 (0.2) | | 0.787 | |
| TEER Tricuspid valve repair (%) | | 2 (0.2) | | 1 (0.2) | | 0.787 | |
| Surgery of ascending aorta (%) | | 1 (0.1) | | 2 (0.3) | | 0.365 | |
| CABG (%) | | 1 (0.1) | | 1 (0.2) | | 0.796 | |
| Surgical atrial septal defect closure (%) | | | 1 (0.1) | 0 (0.0) | | 0.404 | |

CABG: coronary artery bypass grafting; TEER: Transcatheter edge-to-edge.

**Supplemental table S9: Clinical presentation of patients with IE during follow-up**

| **Variables** | | | | | **Automated titanium fastener**  **N = 829**  **(59 %)** | | | **Hand-tied**  **knots**  **N = 576**  **(41 %)** | |
| --- | --- | --- | --- | --- | --- | --- | --- | --- | --- |
| **Patients with IE during follow-up (%)** | | | | 9 (1.1) | | | 17 (3.0) | | |
| **Modified Duke`s Criteria** | | | |  | | |  | | |
| *Major*  *Criteria* | Positive blood culture result (%) | | | | | 7 (0.8) | 15 (2.6) | | |
|  | Positive intraoperative smaer (%) | | | | | 3 (0.4) | 6 (1.0) | | |
|  | Positive imaging findings of IE-related lesion (%) | 9 (1.1) | | | | | | | 17 (3.0) |
| *Minor*  *Criteria* | Cardiac/non-cardiac predisposition (%) | | | 9 (1.1) | | | 17 (3.0) | | |
|  | Fever > 38°C (%) | | | 6 (0.7) | | | 11 (1.9) | | |
|  | Vascular phenomena (%) | | | 4 (0.5) | | | 4 (0.7) | | |
|  | Immunologic phenomena (%) | | 0 (0.0) | | | | 0 (0.0) | | |

Values are presented as prevalence rate, n (%).

IE: infective endocarditis

**Supplemental table S10: Detected pathogens in patients with IE during follow-up**

| **Variables** | | **Automated titanium**  **fastener**  **N = 829**  **(59 %)** | | | | **Hand-tied**  **knots**  **N = 576**  **(41 %)** | |
| --- | --- | --- | --- | --- | --- | --- | --- |
| **Positive blood culture results** | | |  | | | |  |
| Staphylococcus epidermidis (%) | 0 (0.0) | | | | | 3 (0.5) | |
| Staphylococcus aureus (%) | 2 (0.2) | | | | | 3 (0.5) | |
| Streptoccocus mitis (%) | 1 (0.1) | | | | | 2 (0.4) | |
| Enterococcus faecalis (%) | 2 (0.2) | | | | | 1 (0.2) | |
| Escherichia coli (%) | 1 (0.1) | | | | | 1 (0.2) | |
| Staphylococcus lugdunensis (%) | 0 (0.0) | | | | | 1 (0.2) | |
| Enterobacter cloacae (%) | 0 (0.0) | | | | | 1 (0.2) | |
| Psuedomonas aeruinosa (%) | 0 (0.0) | | | | | 1 (0.2) | |
| Caridiobaterium hominis- HACEK group (%) | | | | 0 (0.0) | 1 (0.2) | | |
| Campylobacter species (%) | 1 (0.1) | | | | | 0 (0.0) | |
| Fungi (%) | 0 (0.0) | | | | | 1 (0.2) | |
| **Positive intraoperative smear** | | |  | | | |  |
| Enterococcus faecalis (%) | 1 (0.1) | | | | | 1 (0.2) | |
| Viridans group streptococci (%) | 0 (0.0) | | | | | 2 (0.4) | |
| Caridiobaterium hominis- HACEK group (%) | | | | 0 (0.0) | | 1 (0.2) | |
| Acinetobacter schindleri (%) | 0 (0.0) | | | | | 1 (0.2) | |
| Granulicatella adiacens (%) | 0 (0.0) | | | | | 1 (0.2) | |
| Streptococcus dysgalactiae (%) | 1 (0.1) | | | | | 0 (0.0) | |
| Coxiella burnetii (%) | 1 (0.1) | | | | | 0 (0.0) | |

Values are presented as prevalence rate, n (%).

IE: infective endocarditis
